# Individual Differences and Psychosis-Risk Screening: Practical Suggestions to Improve the Scope and Quality of Early Identification

**DOI:** 10.3389/fpsyt.2019.00006

**Published:** 2019-02-14

**Authors:** Jason Schiffman, Lauren M. Ellman, Vijay A. Mittal

**Affiliations:** ^1^Department of Psychology, University of Maryland, Baltimore County, Baltimore, MD, United States; ^2^Department of Psychology, Temple University, Philadelphia, PA, United States; ^3^Department of Psychology, Northwestern University, Chicago, IL, United States

**Keywords:** individual differences, idiographic, clinical high risk, ultra high risk, prodromal psychosis, early identification, early intervention

## Abstract

Approaches to identifying individuals at clinical high-risk (CHR) for psychosis currently do not carefully weigh considerations around individual differences. Effective identification depends on awareness of factors beyond psychopathology as it is reflected in the current literature, such as sensitivity to idiographic circumstances and individual differences. The inability to address contextual factors when employing the status quo method of identification likely contributes to the unacceptably poor accuracy when identifying people at CHR. Individual differences related to factors such as culture, race, comorbidity, and development likely play an important role in accurate identification, and have the potential to improve the validity of approaches intended to identify this population. Tailored approaches to assessment based on an awareness of context, identity, setting, and preferences of clients are possible, and customizing assessment efforts accordingly may be useful for accurate identification of people at CHR. Highlighting the potential for the existing early identification paradigm to marginalize or misunderstand certain groups, we describe how effective identification and ethical diagnosis require sensitivity to individual differences writ large. We suggest that recognizing the importance of these factors advances a more inclusive and accurate approach to identification.

## Overview

Research related to identifying people at clinical high-risk (CHR) for psychosis has seen exponential growth in the past decade, in part fueled by the building evidence that intervention during this phase can prevent, delay, and/or lessen the severity of future negative outcomes (1, 2). Significant gains in identification include increased accuracy using risk calculators based on large samples and advanced statistical approaches (3–5). Additionally, this body of work has revealed a clinically relevant vulnerability in those at CHR—associated with high rates of substance abuse, trauma exposure, cognitive impairments, and suicidal risk (6, 7)—irrespective of transition to first episode or full-threshold psychosis (8). Findings like these have likely generated a number of recognized and heretofore undiscovered benefits relating to earlier interventions, stronger therapeutic relationships, and shorter duration of untreated psychosis. To build on these gains it is important to consider that while the current CHR assessment approach has relatively adequate positive predictive value in help-seeking individuals, it fails to capture a clinically meaningful percentage of individuals truly at risk for psychosis, and concurrently might over-pathologize individuals who are not at risk. Unfortunately, typical CHR interview practices such as those employed with the Structured Interview for Prodromal Syndromes (SIPS) (9) in North America, may in some cases not fully honor individual differences of those being evaluated for CHR.

In this *Perspective* commentary, we highlight several issues with this “one-size-fits-all” approach in relation to the ethnic and racial differences as well as culture, context and socio-economic status. This has a clear relevance for this special issue on identifying individuals at CHR in different cultures and countries, as we highlight how differences in factors such as race/ethnicity and culture can substantially impact clinician-ascribed diagnostic ratings, even within a single country—a point clearly aligning with the broader volume focusing on cross-cultural and international differences in CHR research. We also discuss how the status quo approach of interviews such as the SIPS' identification of Attenuated Psychosis Syndrome (APS) and related risk states can limit the incorporation of important information relating to comorbidity and developmental considerations, relevant concerns in the CHR population. After highlighting issues for each area, we will discuss suggestions for current research and intervention work, as well as outline a series of goals for future studies in this domain.

## Cultural and Contextual Competence

Evidence suggests that immigrants, ethnic/racial minorities, and those raised in certain urban environments are at a heightened risk for developing psychosis-spectrum disorders (10, 11). It is possible that greater exposure to risk factors for psychosis, including trauma and discrimination associated with any minority identity (e.g., race, sexual identity, gender), lead to higher rates of psychosis symptoms and diagnoses (12). At the same time, it may be that contextual or environmental factors can lead to endorsing items—particularly those related to suspiciousness—on CHR assessment tools when the underlying mechanism is either distally connected or unrelated to psychosis (13, 14). Responses to discrimination, crime, and/or trauma may be causally, concurrently, concurrently that leads to causality, or illusorily linked to psychosis- risk symptoms (13, 14). Further, clinician biases can result in systematically ascribing psychosis-spectrum explanations for culturally distinct behaviors (15). For instance, some common themes in CHR interviews and screening tools probe for very normative behaviors in certain cultures (e.g., belief in superstitions, déjà vu, having special talents, religious convictions). In some cases, endorsement of these more normative prompts is associated with *better* functioning (16). All of these factors can lead to diagnostic confusion, false-positives, and ultimately large-scale health disparities for minority groups.

There are several possible routes to addressing these issues should they arise as consistent concerns in the field. First, it is useful to ensure that interview techniques are sensitive to cultural factors, which may require using structured interviews and potentially modifying existing measures and processes to explicitly probe for these relations. Assessments should allow time for clients to share their individual and cultural views around what are intended as CHR probes, such as their possible experiences of discrimination, social deprivation, and/or trauma related to their surrounding neighborhood context (15, 17). Additionally, screening tools used to indicate risk, and often trigger referrals, should be validated in different cultures and with different racial and minority groups; results should be considered accordingly prior to assuming psychopathology (16). Such analyses could drill down to scale, factor, or the item level. More broadly, designing validation studies to understand the role of other, often related, aspects of identity (e.g., SES, religion/spirituality, cultural identification, help-seeking response style, language differences) can create more individualized approaches to risk assessment. More explicitly infusing cultural competency into risk assessment training (e.g., the role of clinician bias, socially mediated stress as a dynamic factor when establishing risk), and perhaps even empirically measuring assessor's cultural competence may begin to create a more sensitive and possibly accurate workforce (18). These steps may help reduce the risk of misdiagnosis as well as enhance detection in those who may be more vulnerable for risk of a psychotic disorder.

## Comorbidity

Comorbid psychopathology is another key individual difference requiring consideration. In many cases, “CHR symptoms” may more accurately reflect other, non-psychotic psychopathological processes. For example, symptoms of OCD such as recurring thoughts may in some cases resemble unusual thought content in CHR (e.g., “*Have you felt that you are not in control of your own ideas or thoughts?*”), despite being presumed as clinically “distinct.” Additionally, use of psychoactive substances can elicit psychotic-like experiences that persist beyond acute intoxication and therefore be misinterpreted as risk symptoms despite resolving with sustained abstinence. Likewise, the persistent and preoccupying cognitive distortions associated with false perceptions of body image in eating disorders and body dysmorphic disorder can often resemble the delusional thinking and perceptual disturbances experienced in populations at clinical high risk for psychosis (19). Differentiating all of these experiences from “risk for psychosis” vis-a-vis CHR can present challenges, particularly because comorbid health conditions, experiences of adversity, and substance use are not only risk factors for CHR and psychosis, but can also mimic psychosis-risk and appear in conjunction with the CHR state, each of which has different clinical implications (20–23).

There are some practical solutions to addressing comorbidity in those at CHR, should additional research identify comorbidity as a concern for accurate identification of those at risk. Assessing possible contributors to symptoms at the same depth as psychosis risk, in recognition of clinician bias to one's own specialty, can help address whether factors such as trauma, substance use, or eating disorders for instance are contributors, comorbidities, or unrelated to psychosis risk. In many cases, comorbidities can have accompanying psychotic symptoms, suggesting a need in some cases to expand the definition of psychosis in the context of CHR conceptualizations. Additionally, graduate training and continuing education programs providing psychodiagnostic training, periodic re-training, and assessment validity check-ins may limit misdiagnosis or possible assessor drift. Truly attending to these concerns may require more frequent and in-depth follow-up and an openness to new information that may run contrary to initial impressions—efforts that will hopefully provide a more accurate and individualized evaluation. Finally, it is important to recognize that comorbidity is the rule rather than the exception in individuals at CHR. Although CHR status does not appear to reliably predict other outcomes beyond psychosis (24), certainly the rich information regarding comorbidity will serve to strengthen predictive models and relatedly help to better characterize individual variation and thereby promote precision medicine. Notably, these conditions are clinically relevant in their own right, and should be carefully considered and treated in this manner.

## Development

A similar call for developmental considerations can also be made. For instance, pre-adolescents may endorse over valued beliefs (e.g., “*Do you daydream a lot or find yourself preoccupied with stories, fantasies, or ideas?*”), and adolescents may endorse ideas of reference (e.g., “*Have you had the sense that you are often the center of people's attention?*”), when such “symptoms” at face-value are often normative in these age groups (25, 26). Younger individuals might also interpret interview questions in ways that are different from the interviewer's intention or respond in all-or-nothing extremes on self-report measures (26–32). Longitudinal studies of self-reported psychotic-like experiences reveal a declining rate of endorsement with age, suggesting that these experiences may be part of typical developmental processes. Incorporating developmental awareness in our measures and among clinicians will likely increase accuracy of CHR detection (33, 34). For example, it is quite easy for CHR diagnosticians to confuse sensitivity to sound and belief in an invisible audience (experiences that become increasingly sensitive or salient in adolescence) with psychotic experiences (25, 35, 36). Measures of functioning are also likely particularly sensitive to stages of development as well; the functional expectations of adolescents and young adults vary dramatically from year to year.

There are also a number of practical suggestions to address issues around developmental variation in CHR research and treatment, should development be identified as a reliable confound to accurate identification. First, a developmentally-informed conceptualization of risk can be achieved by training clinicians on the unique developmental considerations of this age range, creating anchors within interview tools that reflect typical and atypical development, developing age-informed norms/cutoff scores, maintaining a sensitivity to response style biases, thoroughly probing endorsements to ensure a shared understanding of meaning, and committing to a longitudinal approach (clinically and through research). Second, investigators and clinicians alike can stay current on not only the literature (as we are regularly discovering new potential developmental confounds in this area), but also norms for adolescent behaviors [e.g., around social media use, social engagement patterns, and dating; (37, 38)] and recent trends in subculture identification and practice (a rapidly shifting area with many potential nuances that would likely confound accurate assessment and treatment). Further, investigators can be mindful not to treat the adolescent period as a unidimensional construct, but rather, understand that this is a dynamic span of time, beginning just at the end of late childhood and carrying many individuals into the mid to late 20's. The scope of “normative” behavior, as well as social and role functioning expectations will be much easier to assess with that consideration in mind, and to this point, it may be best to view different stages by the attainment of developmentally relevant landmarks, instead of age or year in school.

## Conclusions

Prevention efforts in psychosis have never been more promising. True prevention will require the CHR concept to expand beyond specialty clinics, perhaps creating meaningful—but not insurmountable—hurdles between the current state of affairs and the aspirations of identifying the large proportion of people at CHR who are currently undetected, and avoiding labeling people as at-risk who are not. Systemic issues, such as increasing education, creating a culture of hope, prevention, and recovery, and reducing stigma so that more people are willing to seek help, are essential to this goal. Other systemic issues include addressing the bifurcation of mental health and education systems relevant to the risk age, engaging community partners beyond mental health specialists, and considering a more pluripotent approach to identification whereby outcomes are more broadly defined beyond the presence of psychosis. Within-field innovations are required as well, such as creating interview tools with increased accuracy and that require less training, and considering mechanisms when building risk models. To reach these goals, we will benefit from methods that require substantial investments, such as diagnostically-fluid prospective studies and studies that incorporate the voices of people at CHR and their families (e.g., participatory action research).

Complementing these initiatives are the promise of contextual and cultural adaptations. In the diagnosis of illnesses with threshold psychosis, for instance, when using the DSM-5 cultural formulation interview to re-evaluate diagnoses in an ethnically diverse sample initially diagnosed by community providers, Adeponle et al. (17) reported that 49% of individuals with initial diagnoses of psychosis were changed to non-psychotic disorders, while only 5% of initial non-psychotic disorders were re-diagnosed as having a psychotic disorder. In the emerging CHR field we argue a similar need to reach beyond nomothetic and normative perspectives, and to peer deeper to consider contextual and individualized approaches to identification and care.

## Author Contributions

All authors listed have made a substantial, direct and intellectual contribution to the work, and approved it for publication.

### Conflict of Interest Statement

The authors declare that the research was conducted in the absence of any commercial or financial relationships that could be construed as a potential conflict of interest.
